# Assisted Reproductive Technologies (ART) and genome editing to support a sustainable livestock

**DOI:** 10.1590/1984-3143-AR2023-0074

**Published:** 2023-09-08

**Authors:** Alejo Menchaca

**Affiliations:** 1 Plataforma de Salud Animal, Instituto Nacional de Investigación Agropecuaria, Montevideo, Uruguay; 2 Fundación Instituto de Reproducción Animal Uruguay, Montevideo, Uruguay

**Keywords:** FTAI, MOET, PIV, sex-sorted semen, CRISPR

## Abstract

This article provides an overview of assisted reproductive technologies (ART) and genome engineering to improve livestock production systems for the contribution of global sustainability. Most ruminant production systems are conducted on grassland conditions, as is the case of South American countries that are leaders in meat and milk production worldwide with a well-established grass-feed livestock. These systems have many strengths from an environmental perspective and consumer preferences but requires certain improvements to enhance resource efficiency. Reproductive performance is one of the main challenges particularly in cow-calf operations that usually are conducted under adverse conditions and thus ART can make a great contribution. Fixed-time artificial insemination is applied in South America in large scale programs as 20 to 30% of cows receive this technology every year in each country, with greater calving rate and significant herd genetic gain occurred in this region. Sexed semen has also been increasingly implemented, enhancing resource efficiency by a) obtaining desired female replacement and improving animal welfare by avoiding newborn male sacrifice in dairy industry, or b) alternatively producing male calves for beef industry. *In vitro* embryo production has been massively applied, with this region showing the greatest number of embryos produced worldwide leading to significant improvement in herd genetics and productivity. Although the contribution of these technologies is considerable, further improvements will be required for a significant livestock transformation and novel biotechnologies such as genome editing are already available. Through the CRISPR/Cas-based system it is possible to enhance food yield and quality, avoid animal welfare concerns, overcome animal health threats, and control pests and invasive species harming food production. In summary, a significant enhancement in livestock productivity and resource efficiency can be made through reproductive technologies and genome editing, improving at the same time profitability for farmers, and global food security and sustainability.

## Livestock to feed people

The second Sustainable Development Goal endorsed by the United Nations pursues the end of hunger, achieving food security and improved nutrition, and promoting sustainable agriculture (UN, 2015). This goal may not be feasible without increasing global meat and milk production, since both are essential sources of nutrients especially for the most vulnerable groups. The growing world consumption of animal proteins is not only associated with the increase in the number of people inhabiting the planet (about 10 million by 2050s), but also by a greater per capita consumption of animal protein over vegetable carbohydrates. People have more access to better quality food, a result of the improvement in developing countries of the gross domestic product and shared prosperity, the reduction of poverty, and the increasing urbanization and globalization. In short, people simply tend to eat more meat as their income increases. Rise in meat consumption also responds to the fact that most people in many cultures find eating meat a tasty and highly desirable experience (Modlinska and Pisula, 2018), enjoying and deriving pleasure from eating beef, lamb or pork. Consumption of meat provided by terrestrial animals grow from approximately 29 kg per person in 1980 to 43 kg in 2020 (FAO, 2023), which is not equally distributed, varying widely between regions from more than 120 kg per person in some countries of the Americas, Europe, Oceania, to less than 20 kg in a number of countries of Africa and Asia. According to the latest FAO projections, meat demand in low and middle-income countries will increase by 200 percent by 2050 (FAO, 2018).

The main consequence of increasing food consumption is the need to increase animal production to feed people, and then increase agriculture or improve pasture and land management to feed animals. Even though meat production has doubled from 1990 to 2020 and increased four-fold since the 1960s, this production is expected to continue to grow for the coming years (FAO, 2018). The challenge of the 21st century seems to be to produce more food without harming - or even improving - global sustainability, which includes different issues such as climate change, deforestation, biodiversity, animal welfare, and animal and human health (i.e., One Health initiative). Thus, the only way forward in the current and future global context is to produce food sustainably, responsibly, and efficiently. Resource efficiency means using the Earth's limited resources in a sustainable manner while minimizing impacts on the environment. It means to produce more with less. On the other hand, if those countries that currently lead the livestock industry do not have the ability to follow this trend, people will push to reduce the consumption of meat and milk, and in the longer-term humans will have changed their eating habits.

This article summarizes the state of different biotechnologies, some already available and others in development, which can make a significant contribution to improve livestock and food supply without negatively altering the planet on which we live.

## Grazing systems for sustainable livestock

Beef is one of the most eaten types of meat worldwide (24% of meat consumption) after pork and chicken (FAO, 2023). The world cattle population is approximately 1.53 billion, of which 35% is in the Americas, 31% in Asia (including buffalo), 24% in Africa, 7.5% in Europe, and 2.3% in Oceania. The major cattle population is located in South America (24%) and more than 75% of these cattle are raised in Brazil, Argentina, Paraguay and Uruguay. These four countries produced approximately 20% of world beef demand in 2021, supplying approximately 37% of frozen and 24% of total world exported beef (FAO, 2023). As a leader in meat and milk production, how are livestock systems managed in this part of the world? And how is the overall efficiency in terms of food production, ecosystem conservation and sustainability?

A large portion of livestock production in South America is based on grazing native rangelands and pastures (Oyarzabal et al., 2020), which includes different vegetation types such as grasslands, shrublands, savannas and meadows, usually distributed in temperate and subtropical regions of the world (Hoekstra et al., 2005; Overbeck et al., 2015). Large herbivores are a key component of such systems (Oesterheld et al., 1999). An important, yet not clearly quantified proportion of the meat consumed worldwide, is produced on these areas. Cattle grazing on rangelands represent one of the few ways to convert low- quality plant material into a high-quality nutritional product for human consumption. Most of the plants that ruminants eat and turn into nutrient-rich meat and tasty food are inedible to humans, and properly managed cattle also represent a sustainable way to produce this widely desired high-quality source of protein. In South America, the Rio de la Plata grasslands (Biome Pampa) occupies approximately 853,000 km^2^ (i.e., part of Argentina, whole Uruguay and South of Brazil), and it represents one of the most diverse, largest, and less transformed grassland area in the world (Oyarzabal et al., 2020). Grazing lands have the highest potential for soil carbon sequestration, reduction of greenhouse gas emissions, water regulation and biodiversity preservation, among the different land cover that occupied the Rio de la Plata grasslands landscapes (Paruelo et al., 2022).

On the other hand, conversely to pasture-based systems, intensive feeding operations such as those conducted in feedlots are associated to great concerns from different perspectives. Raising cattle in feedlots is inherently a very energy inefficient process, since production of the feed, mainly corn, involves large inputs of fossil fuels at every stage from field preparation for planting to harvesting and to transporting the feed to the animals. The next steps in conversion of the high-quality feed by the animals to meat is also inefficient. According to the FAO (Steinfeld et al., 2006), the trophic efficiency of beef production in feedlots ranges from 2% to 7%, which means that only 2% to 7% of the energy in the feed is converted into edible meat. The finishing period in a feedlot represents most of the unsustainable part of beef production. In contrast with the part of the production cycle that take place on grazing lands, intensive livestock systems concentrate gas, liquid and solid emissions, resulting in major point contamination events. Moreover, public opinion is increasingly concerned about the health and welfare of animals raised in concentrated animal operations, and this kind of intensive production system is associated with greater appearance of infectious diseases (some of them affecting humans), indiscriminate use of antibiotics, introducing hormones and growth promoters into the food chain, animal welfare concerns, among others.

In summary, proper rangeland management practices and improved pasture-based systems can expand livestock production, and at the same time maintain ecosystems integrity. In comparison to intensive feeding operations, pasture-based livestock farming is more environmentally sustainable, promotes animal welfare, and provides a kind of meat and dairy products more preferred by consumers.

## Reproductive efficiency, the challenge for the grazing systems

The main challenge of pasture-based systems is the low production efficiency that usually occurs in rangeland and grassland conditions. Low productivity in general is associated with several factors such as rainfall regime variability and forage availability or quality due to seasonal variations. Under these conditions, animals may experience insufficient nutrition during some periods of the year, which can lead to lower weight gain or even worse to significant weight loss, inappropriate animal health and welfare, reduced production traits and impaired reproduction, and in severe cases death of animals. Reproduction is impaired due to a poor health condition characterized by inadequate hormonal function by the hypothalamic-pituitary-gonadal axis, being the anestrus one of the main expression of this dysfunction.

Reproductive efficiency has a major impact on the economic sustainability of a cow-calf operation, since it has 4 to 10 times greater importance than growth and carcass traits (Glaze, 2011; Melton, 1995). As optimum reproductive performance is achieved, other adjustments on the production system may be tuned, having a strong ability to enhance environmental and economic sustainability (Lancaster and Larson, 2022). As discussed above, reproductive performance in grazing systems is associated with delayed puberty in heifers and prolonged postpartum anestrus in suckled cows (prepubertal and postpartum anestrus, respectively). Most of the world’s cow-calf systems intend to obtain an age at first calving of two years for heifers, and to achieve above 90 calves per 100 cows every year. However, in non-intensive cattle systems such as those conducted in many countries worldwide, heifers require 3 or 4 years to produce the first calf in their life, and reproductive rates range from 40 to 80 calves per 100 cows per year. As a consequence, weaning rate in South American countries, except in Uruguay (approximately 65% weaning rate) and Argentina (just over 60% weaning rate), barely exceed 50% (i.e., one calf every two years) and most of them are below this percentage. In short, under these conditions it is necessary to maintain a cow grazing in the field approximately for eight years to get only three calves. Considering that cows have the ability to get one calf per year, the low efficiency in the use of the resources affects not only the economy of the farmers, but also the use of water or land, and the balance on gas emission. According to an estimation model proposed for US beef production system (Davis and White, 2020), the improvement from 50 to 90% in calving rate has significant effect in the environmental impact (i.e., from 30 to 40% in change of land, water, and carbon footprints).

## How to improve reproduction in pasture-based conditions

Transformation of livestock and reorientation of agri-systems is now required as never before, not only from a productive/economical perspective, but mainly for environmental and evolutionary social reasons. The use of certain tools makes this easier by improving animal health, animal feeding and management, genetics and reproduction. Breeding animal technologies have the ability to enhance both genetics and reproductive efficiency. North and South American countries are world leaders not only in beef and milk production, but they are also prominent in development and application of reproductive technologies (Mapletoft et al., 2018). Genetics progress is substantially improved by using these technologies, and productivity is greatly increased simply by producing more calves with the same number of cows. Reproductive technologies such as fixed-time artificial insemination (FTAI), sexed semen, and embryo transfer, noticeably improve the productivity of livestock by allowing to obtain more and better-quality calves in a given period, with the same area and cows. Therefore, this improvement in the efficiency of the herd has a favorable effect not only in the economy but also on the environment, particularly by improving the greenhouse emissions balance. In pasture-based systems, as productivity per hectare increases, the enteric methane emission per unit of meat or milk produced decrease, without significantly increasing emissions per unit area (DeRamus et al., 2003). In other words, the use of these technologies could generate a beneficial impact reducing the environmental footprint of livestock, with low cost and in a short-term period, and at the same time increasing food production.

## Fixed-time Artificial Insemination (FTAI)

In pasture-based systems there is great convenience of well-defined breeding season to adapt different demands of the herd (such as breeding, calving and weaning) to forage availability. Pharmacological protocols for induction and synchronization of ovulation are necessary to produce pregnancies by artificial insemination during a short and predefined breeding season. As farmers face both anestrus and low estrus detection in suckled cows and heifers kept at pasture-based conditions, the FTAI technology has been successfully incorporated in many countries worldwide during the last 20 years (Baruselli et al., 2018; Bó et al., 2018). FTAI has several advantages over traditional long-term insemination schemes based in estrous detection, including the ability to inseminate all cows in the herd at the same time in a single day, reducing labor and management costs associated with estrus detection, and improving reproductive efficiency by reducing the time between insemination and conception. In pasture-based systems, and mainly in rangeland conditions, the breeding objectives are a) to inseminate early in the breeding season, b) to detect early those cows getting pregnant to adjust forage and herd management, and c) to inseminate again as soon as possible those non-pregnant cows that failed at first service. The main obstacle for traditional schemes used in the past based in estrus detection and insemination or natural service, is that most of the suckled cows are in postpartum anestrus and then, without estrous behavior and ovulation, breeding and pregnancy is not possible. At the end of the last century, this problem was overcome by the development of FTAI, and for this reason the adoption of this technology by the private sector dramatically increased from the early 2000s pushing the massive use of insemination in all Latin American countries. Only in the Mercosur region of Brazil, Argentina, Paraguay and Uruguay, insemination has reached approximately 30 million cows per year, and it is growing every year [updated from Mapletoft et al. (2018)]. Of note, in 2000s approximately 2% of the beef cows of these countries received inseminations (most of them after estrous detection), and nowadays approximately from 20 to 30% of beef cows are inseminated every year in each country, mainly by FTAI.

As a result of applying FTAI during 20 years in this region, livestock production was substantially improved. Although it is difficult to have a complete figure of all these countries, at least for those herds that implemented this technology in general the current situation shows that: a) Precocity in heifers was advanced by the pharmacological induction of ovulation and pregnancy in prepuberal females, in some cases allowing to get heifers pregnant at 14 months instead of 2 or 3 years of age; b) Pregnancy rate in both anestrous and cyclic cows was increased in suckled cows, allowing to increase pregnancy rates from 60-70% to 80-90% in two or three months of breeding season; c) Genetic progress in the herds surely was improved in the region, by the use of insemination with selected sires in large scale programs including the whole herd of both heifers and cows; and d) The curve of birth into the calving season was better controlled occurring earlier, by the fact that each cow is pregnant at a prefixed time, considering forage availability and the convenience of the system. This summarizes the main contribution of this technology, currently applied in a considerable percentage of the beef cow population in this region.

## Sexed semen

The production of offspring of a desired sex has great potential to improve productivity of livestock systems but also the resource efficiency. This technology was commercially available for farmers in the early 2000s and in this period has made tremendous contribution in livestock, mainly in dairy cattle. The use of sexed sperm in conjunction with FTAI and *in vitro* embryo production is an efficient manner of obtaining offspring of the desired sex and it allows the dissemination of superior traits in the female or male population without requiring sophisticated techniques or complicate schemes for estrous detection and insemination.

In the case of artificial insemination, sexed semen is used to increase the likelihood of producing female or male offspring, as female cows are generally more valuable for milk production and breeding purposes with lower dystocia (lower birth weight than male calves), while males are more appropriate and efficient for converting grass into meat production. In addition to the enormous advantages of this strategy on the efficiency of livestock, the avoiding surplus male offspring in the dairy industry should be more appropriate regarding animal welfare and euthanasia of low-value male newborns. The management of surplus male dairy calves is an emblematic example in the strategy used by animal rights activists against dairy farming (Weary et al., 2016). Strategic use of sex-sorted semen in conjunction with genomic technologies to identify superior females to satisfy replacement females led to, at the same time, identification of a population of dairy cows from which replacements are not desired, leading to a tremendous increase in use of beef semen in dairy herds. Moreover, it is possible not only to crossbred dairy cows with beef sexed semen to produce male offspring, but to produce only males of selected breeds by using beef sexed semen in conjunction with beef donors for *in vitro* embryo production. In addition, in-house production of replacement females has also implications in animal health control programs by enhancing biosecurity, while numerous other potential applications are evolving or are under consideration (Seidel and DeJarnette, 2022).

This technology is available for producers worldwide, and although pregnancy rate is around 10% lower than that obtained with non-sexed semen and the cost of semen straw is somewhat higher, the economic profit is positive for farmers, with additional advantages for animal welfare, animal health and global resource efficiency and sustainability. Although exact numbers are unavailable, estimations indicate that sex-sorted semen is rapidly approaching 30% of the total insemination market share in North America (Seidel and DeJarnette, 2022). Probably this is not the situation of other countries, but it shows a tendency in the use of this technology that is growing worldwide, mainly in dairy cattle.

During the last years significant improvements were made to this technology, different refinements in sorting equipment and procedures have been done, pregnancy rate has improved even with FTAI, and the price per straw has been slightly reduced. Also, two companies (instead of one) are now in the market and the business model has changed with direct involvement of the genetics industry, further pushing the technology to the market. In the coming years, the implementation of this technology will increase, and in a more long-term period surely it will be adopted by most of the farmers and most of the straws sold will be sexed semen.

## Embryo transfer technologies

Animal genetic progress is substantially accelerated through the use of embryo transfer after superovulation or follicular aspiration (*in vivo* or *in vitro*) from superior females. The production of a large number of embryos per female and the ability to store them for later use and transportation, not only increase the efficiency of breeding programs and the genetic trade, but also reduce the risk of transmission of many pathogens. Emerging, re-emerging and transboundary diseases permanently threat animal health and global meat/milk supply, and the embryo transfer technology is an invaluable tool also for the global health and food security. Production of *in vivo*-derived embryos is a well-established technology applied worldwide since the late 20^th^ century, while *in vitro* embryo production (IVEP) has increased considerably over the past 20 years. Over this period, commercial bovine embryo transfer has become a large business, locally and internationally, for smaller and bigger biotechnology companies. Worldwide, almost 2 million bovine embryos were produced in the last year (both *in vivo*-produced and *in vitro*-derived), of which more than 37% were produced in South America (Viana, 2022), especially in Brazil where almost 700,000 *in vitro* embryos were produced. In the last 20 years genetic improvement programs have benefited significantly from this technology, from 2020 to 2021 total embryo production increased 25%, and in the coming years the contribution of IVEP will surely continue to grow worldwide.

The IVEP has been applied in both beef and in dairy cattle, both to disseminate pure breeds but also to design hybrid schemes by crossbreeding different adaptive breeds, mainly in tropical or adverse conditions. One of the well-known examples is the case of Girolando (Gir x Holstein crossbreds) in dairy cattle, created originally in Brazil and then widely used in many tropical and subtropical countries. This synthetic breed combines the resistance to hot temperatures and to tropical diseases of *Bos indicus* Gyr cattle, with the high milk production traits of Holstein cows, resulting in a more profitable dairy industry in different regions. The association of reproductive technologies such as IVEP from selected high-producing cows, the use of sexed semen to produce female offspring, and the transfer of these crossbred embryos to recipient females of adaptive local or beef breeds, was decisive for the dissemination of these kind of animals in tropical conditions. This breeding strategy is completely available for any farmer and is used in large- and small-scale programs in many countries in the Americas. The example shows how this type of technologies can make a contribution in the production of milk or meat in adverse environments, expanding livestock and food production in a way that has never been possible before.

One of the indirect consequences of the increase of the industry related to IVEP was the increased availability of laboratory infrastructure, as well as of qualified technicians (Viana et al., 2018). This network of laboratories is a platform for the development of other embryo-related technologies, particularly those that require substantial investments in laboratory equipment (e.g., cloning, embryo micromanipulation, genome editing, among others) and have limited commercial demand. In addition, research teams offered by the academic sector acting in close relationship with private companies that in turn are operating with farmers in many countries, generate the conditions to promote and push new technologies to make them accessible to the primary sector.

## Emerging technologies: genome editing

Even though some technologies already available will be implemented and significant improvements in overall livestock efficiency would be achieved, it would not be enough to increase food supply according to the expected consumption, and at the same time enhancing environmental side effects. Conventional strategies that improve animal traits by classical genetic tools such as selecting breeding, even assisted by genomic selection and reproductive technologies, have some biological limitations that cannot be overcome with current tools. Emerging technologies such as CRISPR-based genome editing have the ability to revolutionize biology sciences and can make a great contribution to livestock transformation. Through this biotechnology, it is possible to improve animal production traits, animal health and disease control, animal welfare and resilience, control of pests and invasive species, contributing to maintain animal derived products as the main source of human food. In the meantime, other approaches have been proposed or are being explored such as changing eating habits, developing synthetic foods, and experimenting with different sources of protein including edible insects.

With the CRISPR/Cas system it is possible to edit any given DNA sequence including a single nucleotide, and then, deleting, repairing, or adding particular sequences carrying out different phenotype expression. This strategy merges three different disciplines such as genetic engineering, molecular biology and reproductive technologies, to be applied to different fields from livestock to biomedicine, and in many species from procaryotes to mammals (Menchaca et al., 2018; Mojica and Montoliu, 2016). In addition to introduce alleles from certain animals to others or to shape the genome according to our purpose, genome editing can also be used to rapidly increase the dissemination and frequency of particular alleles in a given population, while simultaneously maintaining the rate of genetic gain in other traits and constraining inbreeding to acceptable levels (Mueller et al., 2019), which is not possible through classical genetic tools.

In the past century, the transfer of genetic material in a controlled and deliberate manner between animals was based on transgenesis, but in general, it was extremely difficult in large animals with many projects being technically infeasible. Since transgenesis implies the insertion of foreign DNA into the host genome, this technology never gained the sympathy of the public opinion. In addition, it requires a hard and long process in the regulatory agencies, which discouraged research and investment. For this reason, the need for an easier, inexpensive, more efficient, and transgenesis-free technology for precise editing of animal genomes was necessary. Nowadays, with the arrival of the CRISPR/Cas system for genome editing, these obstacles were overcome and this technology is now ready to use.

Since 2014, when the first reports with CRISPR/Cas system in large animals were published, the CRISPR/Cas system has revolutionized genome engineering for several species. Unlike transgenesis, genome editing does not necessarily imply the insertion of exogenous DNA into the host genome with substantial advantages for developer companies, regulatory processes, and public opinion. The application of this biotechnology in livestock have been reported first in research projects, then in proof-of-concept studies, and more recently have been proposed for commercial use in market-oriented studies. Different leading genetic companies with a global presence, operating in different species, have announced innovative projects to apply genome editing-based technology to design specific traits in livestock. With the aim to illustrate the ductility and potential of this tool to improve livestock and food production in the current and future global context, some examples are summarized below. For further information specific reviews are recommended (Menchaca, 2021; Tait-Burkard et al., 2018).

### Improved food production

Enhancing animal performance in terms of quantity and quality of food produced is one alternative to improve livestock footprint, by producing more meat or milk without increasing negative environmental effects. At the same time, greater individual production performance increases profitability for farmers, acting as a key factor to involve people that work with animals to accelerate the implementation of this strategy. Several examples of this ability of genome editing to improve yield and quality of food in livestock are available and a couple of cases are described in this section. Multipurpose breeds that efficiently express more than one desirable trait (*e.g*., cows that produce milk and meat, or sheep producing meat, milk and wool) are a rare phenomenon in animal husbandry. For centuries, farmers have used classical approaches like breeding and selection aiming to get closer to this goal. However, multipurpose breeds are often at a disadvantage compared to specialized breeds, and many farmers have ended up accepting that the best decision is to obtain specialized animals for a single trait. This paradigm in livestock may be changed with CRISPR. Sheep with greater growth performance and high-quality wool using CRISPR was one of the first models demonstrated in livestock (Crispo et al., 2015). Double muscle lambs were obtained by producing Superfine Merino lambs carrying a mutation of *MSTN* gene, a mutation that occurs in some meat breeds like Texel and that is difficult to introduce in wool breeds by selecting breeding. The CRISPR-produced lambs achieved greater growth rate that led to a 25% heavier body weight than Superfine Merino lamb counterparts, while maintaining the same wool quality traits. Methods to produce these dual-proposal animals achieved in Uruguay, suggested that what farmers have pursued for centuries might be achieved with CRISPR in only a few months.

Genome editing has been implemented for improving food components affecting consumer health contributing to the Sustainable Development Goals (UN, 2015), by optimizing the use of livestock to feed more people appropriately. For example, healthy milk and dairy products are questioned by some consumers and one of the arguments is related to digestive intolerance and allergy suffered by a certain proportion of people. Beta-lactoglobulin, encoded by the *BLG* gene, is the main component from ruminant milk that can cause an allergic reaction, and researchers in China employed the CRISPR/Cas9 system to produce BLG-knockout goats, successfully abolishing the presence of β-lactoglobulin in milk (Zhou et al., 2017). A similar approach was reported in New Zealand for disrupting *BLG* by genome editing in dairy cows (Wei et al., 2018). These edited animals that produce β-lactoglobulin-free milk provide a novel approach for the safe production of hypoallergenic cows’ milk, which could supply millions of people who suffer from this kind of intolerance with this basic food.

### Animal welfare

Consumers are increasingly concerned with the wellbeing of the animals they eat, and this concern increasingly affects the individual decision of the people and the global business related to diet and eating habits. Innovations that promote animal welfare can play a critical role in consumers decisions and should be considered for sustainability of livestock production. Traditional approaches for improving animal welfare include adapting management and environment to avoid the suffering of animals. Conversely, through genome editing it is possible to improve animal welfare by adapting animals, by avoiding unnecessary invasive or painful practices or by conferring resilience or adaptation of animals to adverse environmental conditions. The concept of welfare-enhanced animals is a novel strategy proposed to avoid animal suffering by designing animal genetics, instead of the endless struggle that imply that people apply certain animal welfare actions. Many routine procedures used in intensive livestock production, such as dehorning in calves, castration in males, tail-docking in dairy cattle, mulesing in sheep, or killing of newborns (or abortion) of an undesired gender, or exposure to environment adverse conditions (e.g., heat stress) often results in immediate or chronic pain. Some of these practices may become unnecessary with the use of CRISPR-based technology.

Horn removal, usually using a hot-iron or chemical burning to destroy the horn-producing cells of the horn bud, induces calf pain and stress, increasingly questioned by consumers. By introgression of the causative Celtic mutation (Pc) into the Holstein cattle genome, the biotechnology company Recombinetics, has produced a polled phenotype of Holstein cows without affecting milking traits (Carlson et al., 2016). Semex, a Canadian-based farmer-owned cattle genetics organization with a global presence, was involved in the development of this strategy and has stated the intention of applying it in the dairy cattle industry worldwide (SEMEX, 2018). Among other examples already reported, the case of resilience to adverse environments to enhance animal well-being also may be substantially improved by copying and pasting specific genetic sequences from naturally adapted or evolved local breeds to intensively selected breeds. Usually, animals are exposed to conditions in nature that can be stressful or hazardous. As an example, heat stress is a considerable limitation for high productivity Holstein cattle that has an impact on animal comfort, welfare, health, reproduction, feed intake and production. The spontaneous “slick” mutation of *PRLN* gene found in the local Caribbean criollo-derived cattle confer a better adaptation to hot climate with superior ability to regulate body temperature during heat stress, improving milk production and reproduction in slick Holsteins compared to wildtype animals (Dikmen et al., 2014; Ortiz-Colón et al., 2018). Through genome editing, this mutation has been copied into the genome of European breed genome cattle, and it was recently approved by FDA for human consumption (FDA, 2022) showing that this technology is close to be implemented commercially. In summary, through applying these strategies novel opportunities to improve animal welfare may be offered, which may encourage public embrace of genome-edited animals to be used in the food supply chain. In addition, it is possible to expand livestock towards adverse environments or increase productivity in current conditions.

### Animal health

Creating virus resistant animals for those diseases that neither treatment nor vaccines are available, or to avoid the use of antibiotics to control certain pathogens that affect livestock, are only some examples of the application of this technology to improve animal health. Pandemics or epidemics are a permanent threat that can have a devastating impact on food supply and economy, including production, industry, and trade of live animals and food. In addition, many animal diseases may be transmitted to humans, with around 60% of current human infectious diseases coming from animals (Yamada et al., 2014). According to Word Organization for the Animal Health, intensification of livestock production, among other factors, predisposes to an unprecedented increase in transboundary, emerging and re-emerging animal diseases and zoonosis (Slingenbergh et al., 2004). Livestock expansion is mainly supported in high-density animal operations, which represents a particular threat for the spread of infectious diseases. As domestic animals are a conduit from wild animals to humans, high density livestock production may facilitate the spread of zoonotic diseases. At the same time, in a context of a more intensive production system, farmers will need to combat diseases facing increased antimicrobial resistance and pressure from consumers to minimize the use of antibiotics, which ultimately also affects public health. In this regard, the relevance of new efforts such as the One Health initiative to attain optimal health for people, animals and the environment, is more and more recognized worldwide. For those countries that are positioned as the global food suppliers for this century, livestock practices should be in accordance with this problematic and looking for innovative solutions.

Generation of disease-resistant animals is a tremendous strategy that was not possible to achieve in the past through conventional tools. In the recent years novel approaches have been proposed through the use of CRISPR/Cas-based system, such as the case of porcine respiratory and reproductive syndrome virus (PRRSV). Economic losses in the pork industry due to this disease account for more than 2.5 billion dollars per year in the US and Europe. Effective methods to control PRRSV have not yet been developed, and depopulation, sterilization, and repopulation seem to be the way to control an outbreak of this virus. By targeting the PRRSV receptor CD163 using CRISPR, resistant pigs to this disease were generated by two different laboratories in the US (Whitworth et al., 2016) and in the UK (Burkard et al., 2018). The CD163 edited piglets were completely resistant to both North American and European PRRSV strains, showing no symptoms and suffering no infection following an *in vivo* viral challenge. This achievement has received financial support from Genus plc (PIC, 2021), an international leader in the porcine and bovine genetics industry with worldwide presence. Based in the use of this tool it is possible to eliminate this persistent disease in few years without sacrificing animals (Petersen et al., 2022), a strategy never implemented before in any other eradication program.

Genome editing has also been proposed to generate pigs resistant to African swine fever (ASF) virus (McCleary et al., 2020; Tait-Burkard et al., 2018), Classical swine fever (CSF) virus (Xie et al., 2018), as well as to increase in cattle their resistance to tuberculosis (Gao et al., 2017). The recent epidemic of ASF has produced massive losses in the pork industry in Asia and Eastern Europe and puts other regions at risk. Researchers from The Roslin Institute in UK have explored a CRISPR-based strategy to reduce the disease expression after ASF virus infection (McCleary et al., 2020; Tait-Burkard et al., 2018). Regarding CSF, other important disease that leads to significant economic losses in the global pork industry, researchers in China reported that CRISPR/Cas edited pigs effectively limited the replication of CSF virus and the disease resistance traits in the founders were stably transmitted to the offspring (Xie et al., 2018). While 100% of the wild type pigs died after a viral challenge with clear symptoms of this disease, all the genome edited pigs remained alive during the experimental period.

Overall, CRISPR appears as a novel strategy to control infectious diseases in livestock by editing single genes involved in the host sensitivity to the pathogen. This kind of application of genome editing provides unprecedented opportunities for the control of classical and new infectious diseases with high impact in animal and public health, contributing to One Health initiative and global sustainability.

### Gene drive for pest control

Avoiding the massive use of pesticides, control the population of disease-carrying arthropods such as ticks, flies or mosquitos, suppress invasive species that harm livestock and agriculture, is a dream of any people involved in agriculture and animal production. Pest species and disease vectors generate significant losses in animal production and the global food supply, since approximately 45% of annual food production is lost due to pest infestation (Sharma et al., 2019). With approximately 4 million tons of pesticides sprayed onto the global landscape each year, the increased use of chemicals to manage this problem could compromise the environment. To worsen the situation, many of the most important arthropod pest species have developed resistance to most of the currently available chemicals used for their control, and safety concerns force the residue limits of pesticides and veterinary drugs in food to be lower and lower when it is not zero tolerance. The result in a medium and long-term period of this trend has been the growth of certain pests in the field, and fewer solutions in the veterinary toolbox to combat them. CRISPR-based strategies hold the potential to control pests and disease vectors without the use of chemicals or pesticides.

Success of spreading a given edited DNA fragment into wild species such as insects or invasive species is completely different than in domestic animals. Whereas in domestic animals a given male carrying the desired trait may be genetically exploited under our desired control by using intensive breeding and reproductive technologies (i.e., artificial insemination and embryo transfer), in wild species any trait carried by few males introduced into the population may be rapidly neutralized and disappear due to the natural mechanisms of Mendelian inheritance. To avoid effect exerted in wild populations, the plasticity of CRISPR/Cas system described above has been adapted to create a “driving mechanism”, named gene drive, to increase the chances of the edited trait being passed on to all the offspring particularly required in a wild population. This mechanism overcomes the Mendelian inheritance, where one copy of a gene has a 50% chance of being included in any gamete, and conversely, with gene drives it can be present in a higher frequency (up to 100%) of the offspring. Therefore, gene drives can rapidly spread into a given population, with the potential that after releasing relatively few individuals containing the gene drive, the entire population can incorporate it within several generations.

How could CRISPR-based gene drive systems be used to support other efforts behind global sustainability? After identifying and modifying by CRISPR/Cas system those genetic determinants of key traits such as reproduction fitness, male:female ratio, or certain characteristics required to remain viable in nature, the species is suppressed and finally tends to disappear. Various pests, invasive species, and disease vectors have been proposed as potential applications for their control by this strategy, mainly in the context of conservation efforts done on some fragile ecosystems, and even carried out by some leading livestock countries such as New Zealand and Australia (Dearden et al., 2018; Moro et al., 2018). Application of this tool transcends livestock production, and includes potential benefits for the environment, as well as animal and public health. An example of how this technology may be applied to control a vector borne disease is the case of controlling mosquitos to eradicate malaria in Africa. This project is one of the most advanced gene drive initiatives and is being used as reference for forthcoming projects in other species. The control of malaria has been a global public health priority for almost 100 years, and pioneering studies demonstrated that this technology has the potential to achieve mosquito population suppression (Kyrou et al., 2018; Simoni et al., 2020) or to alter its capacity to transmit pathogens causing the disease (Carballar-Lejarazú et al., 2020; Pham et al., 2019). Vector-borne diseases have broad relevance for both human health and livestock production, since the same vector-pathogen system often affects human and animal health, and in some cases multiple host species can play epidemiologically significant roles. According to WOAH, a full one-quarter of the terrestrial vertebrate pathogens are vector borne and in addition to the example of malaria, CRISPR-based strategies may confer different traits to other vector species to control diseases in animals and humans, such as dengue, Zika virus, chikungunya, yellow fever, trypanosomiasis, leishmaniasis, Chagas, and Lyme disease, among others. Various species of mosquitoes, flies and ticks generate significant losses in food production as vectors of different diseases or directly by their action on the host species. CRISPR-based strategies are being conducted in Uruguay to control *Cochlyomia hominivorax* (Fresia et al., 2021), a fly that produces screwworm myasis in warm blooded species causing suffering and death in affected animals, and producing economic losses in livestock in all the South American countries of about 3.6 billion dollars each year (Vargas-Terán et al., 2005).

After insects, vertebrate invasive species are likely to be the next target for CRISPR-based strategies in wild populations. Invasive species are often introduced into new environments where they become established and cause harm to livestock, human health and the environment. Environmental degradation, threatening of local biodiversity, extinction of native wildlife species, and agricultural or livestock losses are consequences often incurred in different regions after invasive species have been introduced. The Global Invasive Species Database published by the International Union for Conservation of Nature (GISD, 2021) recognizes thousands of invasive species, with some species of rats, rabbits, squirrels, wild boars, possums, foxes, and cane toads among the top 100 of the world’s worst invasive alien species whose population should be controlled. While important efforts to manage invasive species are ongoing (e.g., shooting, poisoning, trapping, or killing with other predators), current methods are usually primitive, costly, have varying levels of efficacy at low population densities, are often associated with unacceptable suffering for target and non-target species, may have unintended ecological consequences, and are usually limited to short-term results in population control (Moro et al., 2018). Gene drives are now being discussed as tools to reduce invasive animal populations or to aid threatened species, since it may offer a more cost-effective, humane, and species-specific alternative to current approaches (Esvelt et al., 2014). However, although successful in mosquitos and some other insects, technical challenges exist for the adaptation of this technology into vertebrates, actively being studied in various laboratories worldwide.

## Concluding remarks

One of the biggest challenges facing humans in this century is increasing food production to guarantee food security in an equitable manner, and even more difficult achieve it without negatively affecting the environment, maintaining the planet's biodiversity, respecting animal welfare, and ensuring animal and public health. The only way to reach this goal is by transforming livestock production. Pasture-based production systems should be better managed to improve resource efficiency and food productivity. Certain biotechnologies are already available, such as FTAI, sexing semen and *in vitro* embryo production, which increases the number of desired animals produced per 100 cows and improves genetic traits more suitable for the industry, producing thus more food. Although considerable livestock improvement would be achieved through these well-established technologies, additional changes should be implemented. Emerging technologies like CRISPR-based genome editing are also available with the ability to do a greater contribution, however, it requires further implementation and investment to be applied worldwide. Only through this type of transformation of livestock will it be possible to produce – in a responsible and environment friendly manner - more meat and dairy products to feed people, complying with the global sustainable goals for this century.
